# Rho Kinase Inhibitor Y27632 Improves Recovery After Spinal Cord Injury by Shifting Astrocyte Phenotype and Morphology via the ROCK/NF-κB/C3 Pathway

**DOI:** 10.1007/s11064-022-03756-0

**Published:** 2022-09-14

**Authors:** Yongyuan Zhang, Xiaohui Wang, Chao Jiang, Zhe Chen, Shuangyang Ni, Hong Fan, Zhiyuan Wang, Fang Tian, Jing An, Hao Yang, Dingjun Hao

**Affiliations:** 1 Xi’an Jiaotong University Health Science Center, 710000 Xi’an, China; 2 Department of Spine Surgery, Hong Hui Hospital, Xi’an Jiaotong University, 710054 Xi’an, China; 3 Xi’an Medical University, No.74 Han’guang North Road, Beilin District, Xi’an, Shaanxi Province China; 4 Department of Neurology, The Second Affiliated Hospital of Xi’an Jiaotong University, 710004 Xi’an, China; 5 Translational Medicine Center, Hong Hui Hospital, Xi’an Jiaotong University, 710054 Xi’an, China

**Keywords:** Rho kinase inhibitor, Spinal cord injury, Astrocyte, NF-κB, C3, S100A10

## Abstract

Spinal cord injury (SCI) usually results in loss or reduction in motor and sensory functions. Despite extensive research, no available therapy can restore the lost functions after SCI. Reactive astrocytes play a pivotal role in SCI. Rho kinase inhibitors have also been shown to promote functional recovery of SCI. However, the role of Rho kinase inhibitors in reactive astrocytic phenotype switch within SCI remains largely unexplored. In this study, astrocytes were treated with proinflammatory cytokines and/or the Rho kinase inhibitor Y27632. Concomitantly the phenotype and morphology of astrocytes were examined. Meanwhile, the SCI model of SD rats was established, and nerve functions were evaluated following treatment with Y27632. Subsequently, the number of A1 astrocytes in the injured area was observed and analyzed. Eventually, the expression levels of nuclear factor kappa B (NF-κB), C3, and S100A10 were measured. The present study showed that the Rho kinase inhibitor Y27632 improved functional recovery of SCI and elevated the proliferation and migration abilities of the astrocytes. In addition, Y27632 treatment initiated the switch of astrocytes morphology from a flattened shape to a process-bearing shape and transformed the reactive astrocytes A1 phenotype to an A2 phenotype. More importantly, further investigation suggested that Y27632 was actively involved in promoting the functional recovery of SCI in rats by inhabiting the ROCK/NF-κB/C3 signaling pathway. Together, Rho kinase inhibitor Y27632 effectively promotes the functional recovery of SCI by shifting astrocyte phenotype and morphology. Furthermore, the pro-regeneration event is strongly associated with the ROCK/NF-κB/C3 signal pathway.

## Introduction

Spinal cord injury (SCI) is a devastating neurological disorder caused various traumas or diseases with high mortality and disability rates. According to the Global Burden of Disease Study, the age-standardized incidence rates of spinal cord injury (SCI) is 13 /100,000 and shows a continuous upward trend worldwide[1]. SCI usually results in cellular damage and loss, as well as inflammatory infiltrates and the formation of compact glial scars[2], therefore, leading to a permanent loss or reduction in motor and sensory functions. Astrocytes constitute the most abundant cell type in the spinal cord[3]. Rudolf Virchow made the first description of glial cells in the mid-19th century[4]. Astrocytes play an crucial role in numerous neural activities such as supplying energy to neurons, neurotransmitter metabolism, neurotrophic support, synaptogenesis, blood-brain barrier maintenance, and immune modulation[5]. When the CNS suffers diverse insults such as injury, degeneration, and infection, astrocytes will rapidly respond to all forms of CNS insults, leading to a change in a variety of protein expression, morphology, and function, known as astrocyte reactivity[6]. Intriguingly, reactive astrocytes exert a dual role in SCI. The detrimental effects primarily manifest in aggravated neuroinflammation and hindered synapse sprouting or axon growth. However, the beneficial roles are associated with anti-inflammation, neuroprotection, and blood-brain barrier repair[7], which largely depends on the phenotypes of the reactive astrocytes. Notably recent studies revealed that depending on their expression profile the activated astrocytes can be divided into two subtypes A1 or A2 reactive astrocytes. The A1 reactive astrocytes are neurotoxic while the A2 astrocytes are neuroprotective[8–15]. Consequently, inducing A2 astrocytes or inhibiting A1 astrocytes after SCI is essential for neuron survival, axonal regeneration, and subsequent functional recovery.

RhoA, a member of Rho-family small GTPases, is a key regulator of cytoskeletal and cell adhesion dynamics and controls a wide range of cellular processes, including morphogenesis, migration, proliferation, and survival[16]. ROCK serves as a major downstream effector of RhoA. Moreover, once the RhoA/ROCK pathway is activated it results in neuronal apoptosis, neuroinflammation, blood-brain barrier dysfunction, astrogliosis, and axon growth inhibition within SCI[17]. Interestingly, several nerve growth inhibitory molecules produced after SCI act on the RhoA/ROCK signal pathway through complex signal transduction pathways, resulting in the reorganization of the cellular actin skeleton; thus, affecting axon regeneration which is not conducive to the nerve function recovery of SCI[18]. Accordingly, suppression of the RhoA/ROCK signal pathway is likely advantageous for neural regeneration. In numerous studies, Y27632, a ROCK inhibitor, was reported to foster neurological improvement after a SCI[19, 20]. Additionally, the activation of the RhoA/ROCK signal pathway enhanced CRMP-2 phosphorylation leading to growth cone collapse, and phosphorylated cytoskeletons-associated proteins to inhibit axon regrowth[16, 21]. Conceivably, the neuroprotective effect of Y27632 is directly related to the mechanisms mentioned above. However, the relationship between Y27632 and the phenotype of reactive astrocytes involved in SCI remains unclear.

NF-κB is a key transcription factor that mediates inflammation and other complex biological processes. The activation of NF-κB can upregulate complement 3 (C3) release of the astrocytes[22], and the increase in C3 transcripts was positively correlated to motor disability[23]. Furthermore, NF-κB transcription activity in glial cells is augmented by the activation of RhoA/ROCK[24–30], leading to acute inflammatory injury. Nevertheless, the precise molecular mechanisms underlying RhoA/ROCK, NF-κB, and C3 in regulating reactive astrocyte phenotype in SCI still need to be further studied.

In the present study, we established an in vitro model of astrocyte culture and a rat model of SCI to investigate the effects of the ROCK inhibitor Y27632 on the transformation of the naive astrocytes and A1 phenotype reactive astrocytes into A2 phenotype reactive astrocytes. We further elucidated the potential mechanisms of repression of RhoA/ROCK as well as down-stream signaling pathways involved in functional recovery of SCI and the intricate switch of astrocyte phenotypes.

## Materials and Methods

### Experimental Animals and Ethical Statements

Healthy Sprague-Dawley (SD) rats (female, 2.5 months old, body weight 150–200g) were used as the SCI model. Neonatal SD rats (24h) used for the primary cell culture of astrocytes were provided by the Animal Feeding Center of the Xi’an Jiaotong University Health Science Center (Xi’an, SN, China). All experimental procedures were performed according to the Guide of Laboratory Animal Care and Use from the United States National Institutes of Health and were approved by the Institutional Animal Care and Use Committee (IACUC) of the Xi’an Jiaotong University.

### Materials and Reagents

Dulbecco’s modified Eagle’s medium (DMEM)/F12, fetal bovine serum (FBS), trypsin, phosphate buffer saline (PBS) were purchased from Gibco (Carlsbad, CA, USA); penicillin G, streptomycin, glutamine, poly-Lysine (PLL), ethylenediaminetetraacetic acid, Interleukin-1α and bovine serum albumin were purchased from Sigma-Aldrich (St. Louis, MO, USA); Y27632, C1q native protein and anti-C3 antibodies were purchased from Abcam (Cambridge, MA, USA); S100A10 Monoclonal Antibodies were purchased from Thermo Fisher Scientific (Waltham, MA, USA); TNF-α and pNF-κB were purchased from Cell Signaling Technology (Danvers, MA, USA); Fluor594-conjugated donkey anti-chicken, and Fluor488-conjugated goat anti-mouse IgG were purchased from Molecular Probes (Eugene, OR, USA); Cell Counting Kit-8 was purchased from Boster (Wuhan, China); Cell culture plates, plastic coverslips, dishes, and flasks were all purchased from Corning (Corning, NY, USA).

### Primary Culture and Purification of Astrocytes

Primary astrocytes were prepared and purified according to our previously reported methods[31] with minor modifications. Briefly, cortices were dissected from postnatal rats (days 1–3). The white matter and meninges were removed under a dissecting microscope, then the rest were minced and digested with 0.25% trypsin for 20min. Digestion was terminated using DMEM/F-12 containing 10% fetal bovine serum. After centrifugation at 1,000rpm for 5min, the cells were resuspended and maintained on uncoated culture dishes in a 37 °C, 5% CO_2_ incubator for 30min. The non-adhesive cells were collected, spun down, resuspended in DMEM/F-12 cell culture medium containing 10% fetal bovine serum supplemented with 1% Penicillin/Streptomycin (adjusted the cell concentration to 1 × 10^5^/mL by cell count), seeded onto poly-L-lysine (PLL, 0.1mg/mL) pre-coated flask and maintained for 10 days to reach a confluence with minimal changes every 3–4 days. To remove the oligodendrocyte precursors and microglia, the flasks were washed twice with PBS, shaken cross in hand at room temperature (RT) for 5min with 0.05% trypsin, and then washed twice with PBS again. Subsequently the cells were detached with 0.25% trypsin and sub-cultured into cell flask and glass coverslips to identify the cell purity. When the purity of astrocyte reached over 95% the cells were used for further downstream experiments.

### Treatment of Astrocytes

To examine the effects of Y27632 on the switch of astrocyte phenotypes, the astrocytes were sub-cultured onto coverslips, 35-mm dishes, 6-well plates, and 96-well plates. The cells were then divided into the following groups: (1) naive astrocytes; (2) astrocytes treated with IL-1α (3 ng/mL) + TNFα (30 ng/mL) + C1q (400 ng/mL); (3) astrocytes treated with Y27632 (10 µM); (4) astrocytes treated with IL-1α (3 ng/mL) + TNFα (30 ng/mL) + C1q (400 ng/mL) and Y27632 (10 µM). Next, 24-72h later the cells were processed for the downstream experimentation.

### CCK-8 Assay

Cell proliferative was evaluated using a cell counting kit-8 (CCK-8) assay. Briefly, after the astrocytes underwent various treatments as mentioned above, the cell suspension was collected and 100 µL of the suspension was added to the 96 well plates at a density of 1 × 10^4^ cells per well. After incubation at 37 ℃ for 2h, the medium containing 10% CCK8 was added to the cell cultures and incubated for 2h. The absorbance, expressed as the optical density (OD), was determined on a spectrophotometer at 450nm for the measurement wavelength. The experiment was repeated in triplicate.

### Evaluation Astrocyte Morphology

Seventy-two hours after treatment the cell-bearing extensions were evaluated using a phase contrast microscope with a 20-fold magnification. At least 15 cells per view were randomly chosen to count. A total of 10 areas were used for each group. The length of the extensions was determined using a confocal epifluorescence microscope (Leica, Wetzlar, Germany). Notably, the average process length is characterized as the average of the astrocytes’ leading and trailing processes.

### Scratch Wound Assay

To analyze the migratory capacity of astrocytes undergoing different treatments, the scratching astrocyte monolayer method was used to examine the astrocyte migratory ability[32]. Briefly, 500,000 astrocytes were transferred into the wells of a 6-well plate and cultured for at least 24h until cell confluence reached greater than 99%. The confluent astrocyte monolayer was scratched in a straight line with a sterile pipette tip (200 µL) following the marker guide sheet under the 6-well plates. The cells were washed 3 times with PBS to remove the detached cells and debris. Concomitantly, the astrocytes were treated with 1% FBS-DF12 containing IL-1α (3 ng/mL) + TNFα (30 ng/mL) + C1q (400 ng/mL), Y27632 (10 µM), and IL-1α (3 ng/mL) + TNFα (30 ng/mL) + C1q (400 ng/mL) + Y27632 (10 µM). Next, bright-field images (magnification 10×) of cells were captured at 0, 6, 12, and 24h after the scratches were made. The ratio of cell migration was calculated as the percentage of the remaining cell-free area against the initial scratch area. At a 10×scaling, a contour was drawn around the cells at the edge of the scratch and the wound area was calculated in square microns.

### Establishment and Intervention of a Spinal Cord Injury Model in Rats

The modified rat SCI model was established as described previously[33]. Briefly, eighteen adult SD rats were randomly divided into three groups: Sham group, SCI group, and Y27632 group. Rats were anesthetized intraperitoneally with 1% sodium phenobarbital (40mg/kg). The skin overlying the T2–12 vertebrae was shaved and disinfected with 10% iodophor and 75% alcohol. A laminectomy was performed to expose the spinal cord from T9 or T10 for all groups. The spinal cord was clamped using an the improved parallel-moving clip compression designed by our lab for 25s. For the sham group, no injury was delivered. Concomitantly, the Y27632 group was injected with Y27632 (0.1mg/kg) intraperitoneally every 24h, while the other groups were injected with the same volume of saline[34]. After surgery, two rats were randomly placed in each cage where penicillin (1 × 10^4^ U) and gentamicin (8 × 10^4^ U) were administered to each rat subcutaneously for 3 days to prevent postoperative infection and buprenorphine allowed for potent for analgesia to reduce pain. One week after the operation, the section of the spinal cord containing the injury site was then obtained for tissue sectioning.

### Basso–Beattie–Bresnahan Locomotor Rating Score (BBB Score)

Functional analysis was assessed using the Basso, Beattie, and Bresnahan (BBB) locomotor scale according to previously established behavior tests[35]. Briefly, The BBB scores of all groups were recorded 1h before surgery, and then at 1d, 3 d, 7 d, 14 d, 21 d and 28 d post-surgery. The BBB scores for each group were observed and recorded by two trainees blinded to the experimental group allocation, and then averaged.

### Immunofluorescence Staining

Astrocytes that were seeded onto plastic coverslips of all groups were washed 3 times with PBS, fixed with 4% paraformaldehyde for 30min, treated with 3% BSA in 0.01M PBS for 30min, and incubated with primary chicken monoclonal antibodies against GFAP and rabbit monoclonal antibodies against C3/S100A10 at 4°C overnight. After washing in PBS 3 times for 5min, coverslips containing the cells were incubated with the corresponding fluorescence conjugated secondary antibodies for 2h followed by DAPI nuclear staining at RT for 10min. The coverslips were then inverted onto the glass slides and sealed using an antifading mounting medium. The cells of all groups were treated independently and analyzed in triplicate.

At 7 days post-surgery, rats of all groups were anesthetized with 1% sodium phenobarbital (4 mL/kg), and transcardially perfused with 0.9% saline and fixed using 4% paraformaldehyde. Subsequently, the spinal cords were dissected, post-fixed in 4% paraformaldehyde overnight at 4°C and soaked in 30% sucrose until the tissue was no longer buoyant. Moreover, frozen sections with a 10μm thickness were prepared and pasted onto slides for immunofluorescent staining analysis. Immunofluorescence staining of the spinal cord sections was conducted the same way as the cell staining procedure. All slides were observed under a Leica DM6 B microscope (Leica Microsystems, Germany). All images were captured using the Leica LAS X software (Leica Microsystems, Diegem, Belgium), and the results were measured with Image J software.

### Western Blot

The protein extracted from the cells with different treatments was collected, and western blotting was performed according to the protocol described previously[31]. Primary antibodies against the following proteins were used: C3, ROCK2, NF-κB, pNF-κB, and β-actin. All primary antibody dilutions were applied according to the manufacturer’s instructions. β-actin was used as the internal control. Immunoblots were visualized using enhanced chemiluminescence reagents. Densitometric analysis of the bands was repeated 4 times and an integrated densitometry value (IDV) was calculated.

### Statistical Analysis

For all analyses, data are expressed as mean ± standard deviation(SD)from at least three independent experiments. Statistical analysis was performed using the statistical software SPSS 26.0. ANOVA was used to analyze significant differences among groups under different conditions, and *P* < 0.05 was considered statistically significant.

## Results

### Characterization and Purification of Astrocytes

**Fig. 1:**
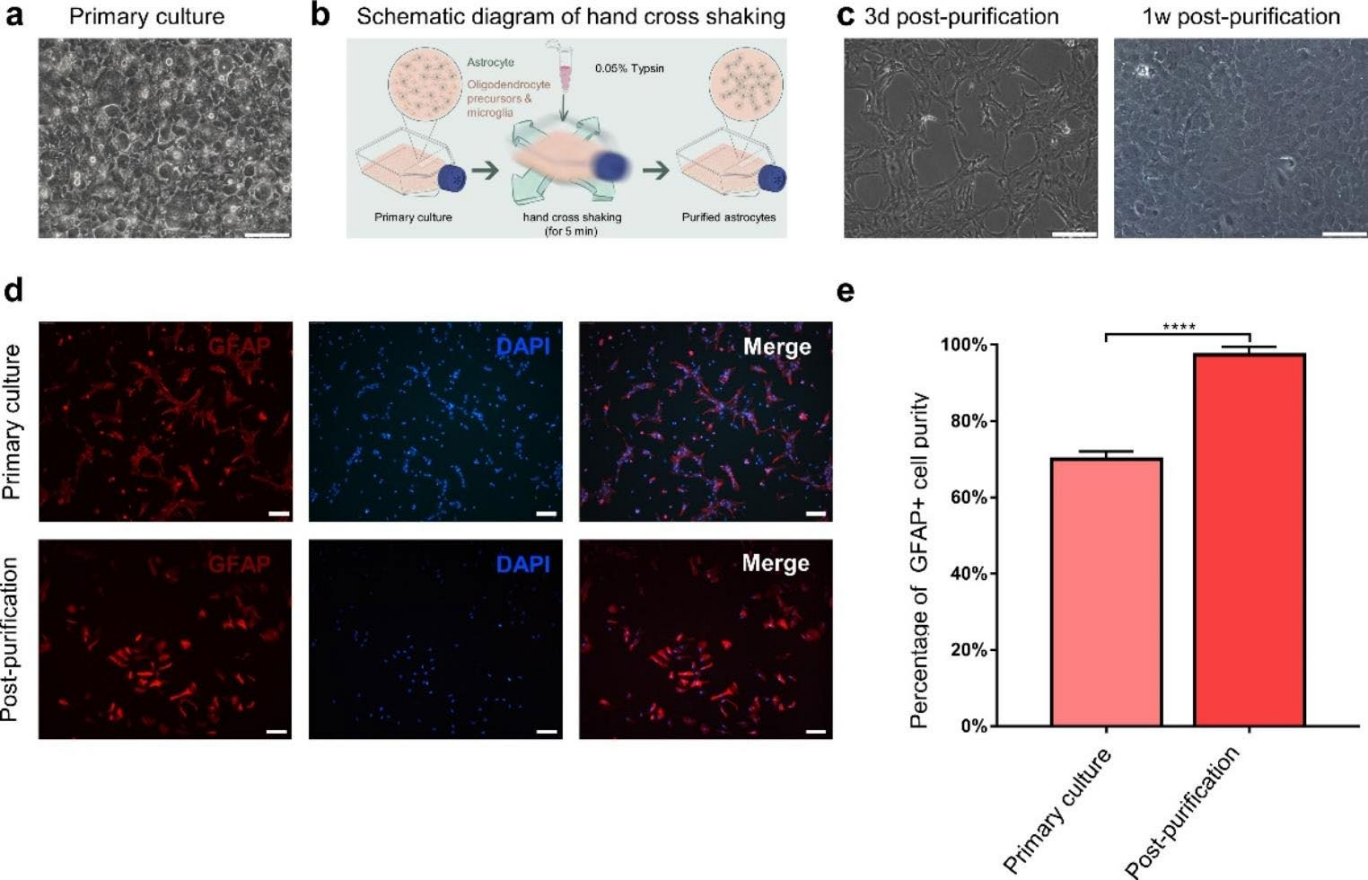
The primary culture and purification of astrocytes. (**a**) Bright field of astrocytes with primary culture. (**b**) A schematic diagram depicting the method of purification by hand cross shaking at RT. (**c**) Bright field of astrocytes post-purification. (**d**) Immunofluorescence identification of astrocytes (GFAP+) showed that the percentage of GFAP + cells post-purification was higher than primary culture (*P* < 0.0001). Scale bars, 100μm

To investigate the effects of Y27632 on astrocytes, astrocytes were first cultured as described in the methods section. As shown in Fig.1, the large majority of cells showed a flat and polygonal morphology and reached more than 99% confluence after 10 days of incubation (Fig.1a). 3–5 days after purification by hand cross shaking at RT (Fig.1b), the purified astrocytes exhibited an irregular shape with processes and gradually became confluent. No apparent oligodendrocyte precursor-like and microglia-like cells with small soma and short processes over the astrocytes were seen (Fig.1c). In addition, immunostaining with glial fibrillary acidic protein (GFAP) demonstrated that there are a large number of GFAP-negative cells prior to purification while the GFAP-negative cells accounted for a very low proportion of the cellular population after purification (Fig.1d). Quantitative analysis revealed that the purity of astrocytes during primary culture (GFAP-positive, 70 ± 1.16%) was lower compared to post-purification (GFAP-positive, 97.33 ± 1.20%) (Fig.1e) (*P* < 0.0001).

### Y27632 Induced Extensions in Astrocytes

**Fig. 2:**
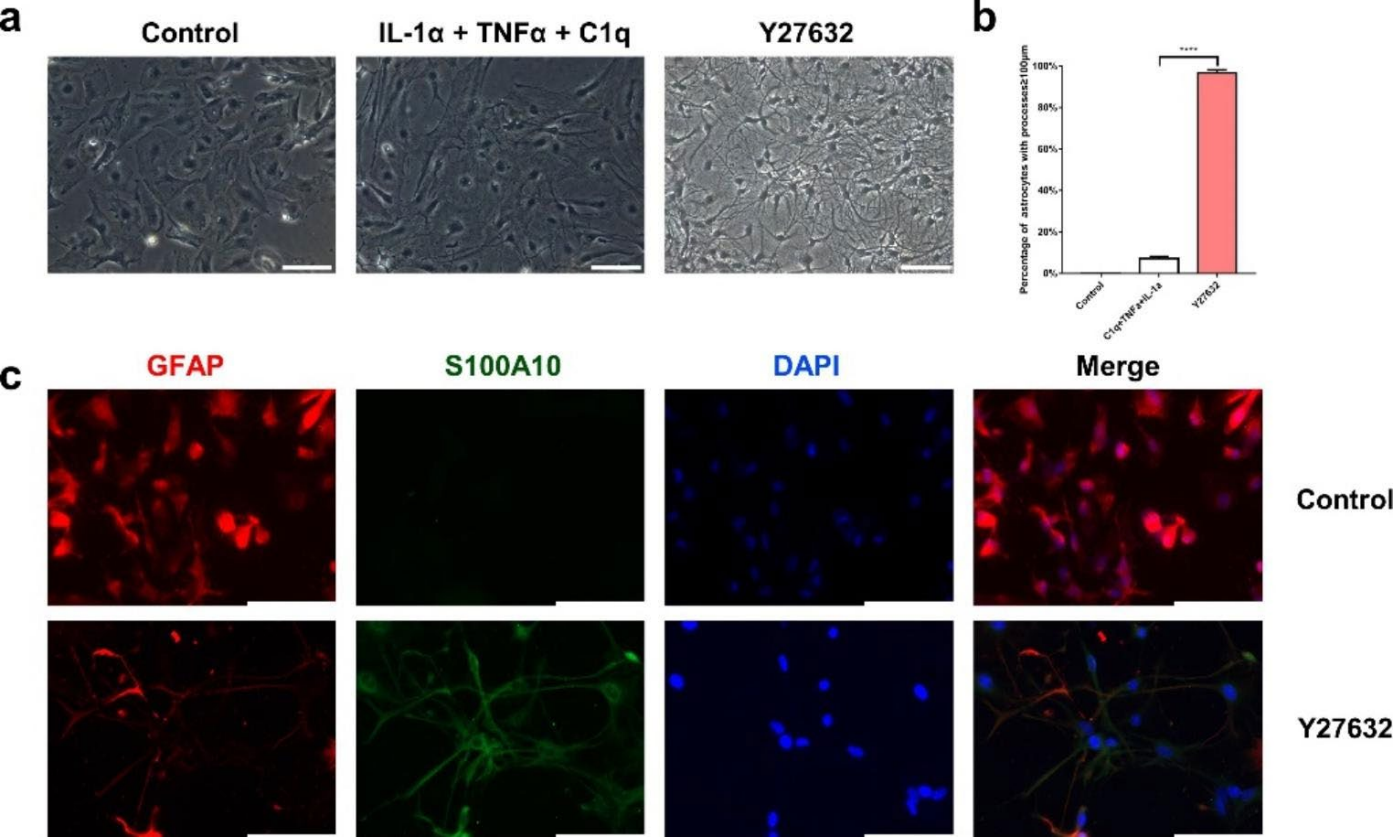
Y27632 induced the shift of morphology of astrocytes. (**a**) Phase-contrast microscope showed the cells of control groups had a flattened shape, while the cells treated with Y27632 exhibited a process-bearing shape. (**b**) 96.67 ± 0.01% of the cells treated with Y27632 developed more than two processes that were ≥ 100μm. (**c**) Immunofluorescence showed that the processes of the cells treated with Y27632 were GFAP^+^/S100A10^+^. Scale bars, 100μm

The administration of the ROCK inhibitor Y27632 caused pronounced changes in astrocyte morphology. As shown in Fig.2, the majority of astrocytes treated without or with IL-1α + TNFα + C1q had a flattened shape and were S100A10-negative, while almost all astrocytes treated with Y27632 exhibited a process-bearing shape under the phase-contrast microscope and were S100A10-positive (Fig.2a and c). Consistent with the morphological observations and immunofluorescence, quantitative analysis showed that after administration of Y27632, approximately 90% of the cells developed more than two processes that were ≥ 100μm (Fig.2b). In contrast, the addition of IL-1α + TNFα + C1q did not develop the cellular characteristics. There were significant differences between the control groups and the Y27632 groups (*P* < 0.0001).

### Y27632 Potentiated the Proliferation and Migration Abilities of the Astrocytes

**Fig. 3:**
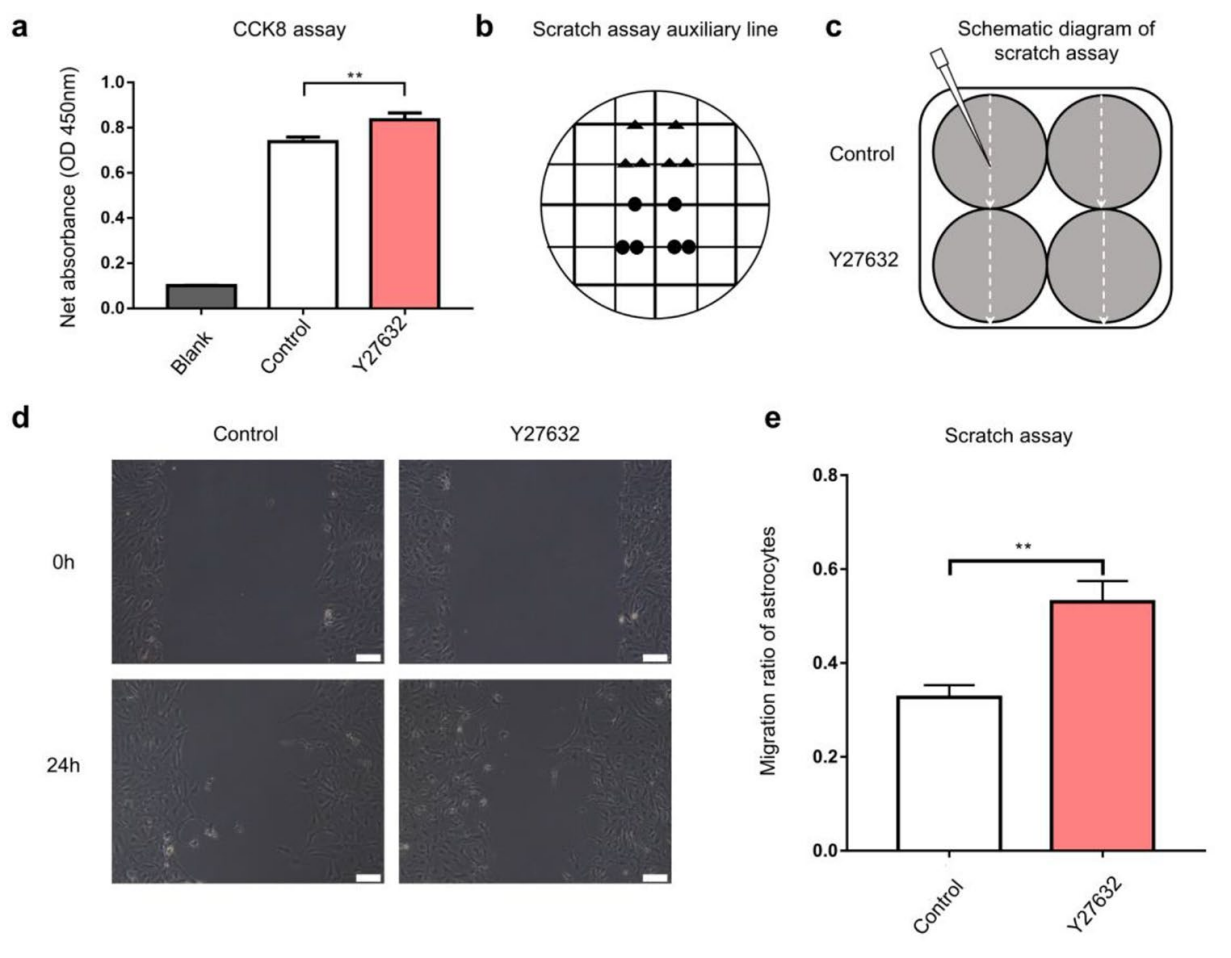
Y27632 increased the proliferation and migration abilities of the astrocytes. (**a**) The CCK8 assay showed that the Y27632 improved the proliferation of astrocytes. (**b**) The auxiliary lines assisted to the wound scratch. (**c**) A schematic diagram depicting the scratch assay. (**d**) The astrocytes were scraped by pipette tip (200 µL), and the final scratch areas (24h later) were calculated. (**e**) The migration ratio of astrocytes treated with Y27632 was higher compared to the control group (*P* < 0.01). Scale bars, 100μm

In addition to the characteristic extension in astrocytes, elevated proliferation, and migration ability are crucial hallmarks of activated astrocytes. Thus, astrocyte proliferation and migration was assessed using CCK-8 and scratch wound assays, respectively. The results revealed that the Y27632 treated astrocytes showed an elevated astrocytes proliferation (*P* = 0.0017, Fig.3a). The wound was created by scraping in a straight line with a sterile pipette tip (200 µL) across the surface of confluent astrocyte monolayers (Fig.3b and c) and then incubated for 24h; the free image areas were all reduced in the astrocytes treated without or with 10 µM Y27632. However, the larger free image area was reduced in astrocytes treated with 10 µM Y27632(*P* = 0.0024, Fig.3d and e) compared to the astrocytes that were only incubated in 1% FBS-DF12 media. These results suggested that the Y27632 increases the proliferation and migration abilities of the naive astrocytes.

### Y27632 Transformed the Naive Astrocytes and the A1 Reactive Astrocytes into A2 Reactive Astrocytes in Vitro

**Fig. 4:**
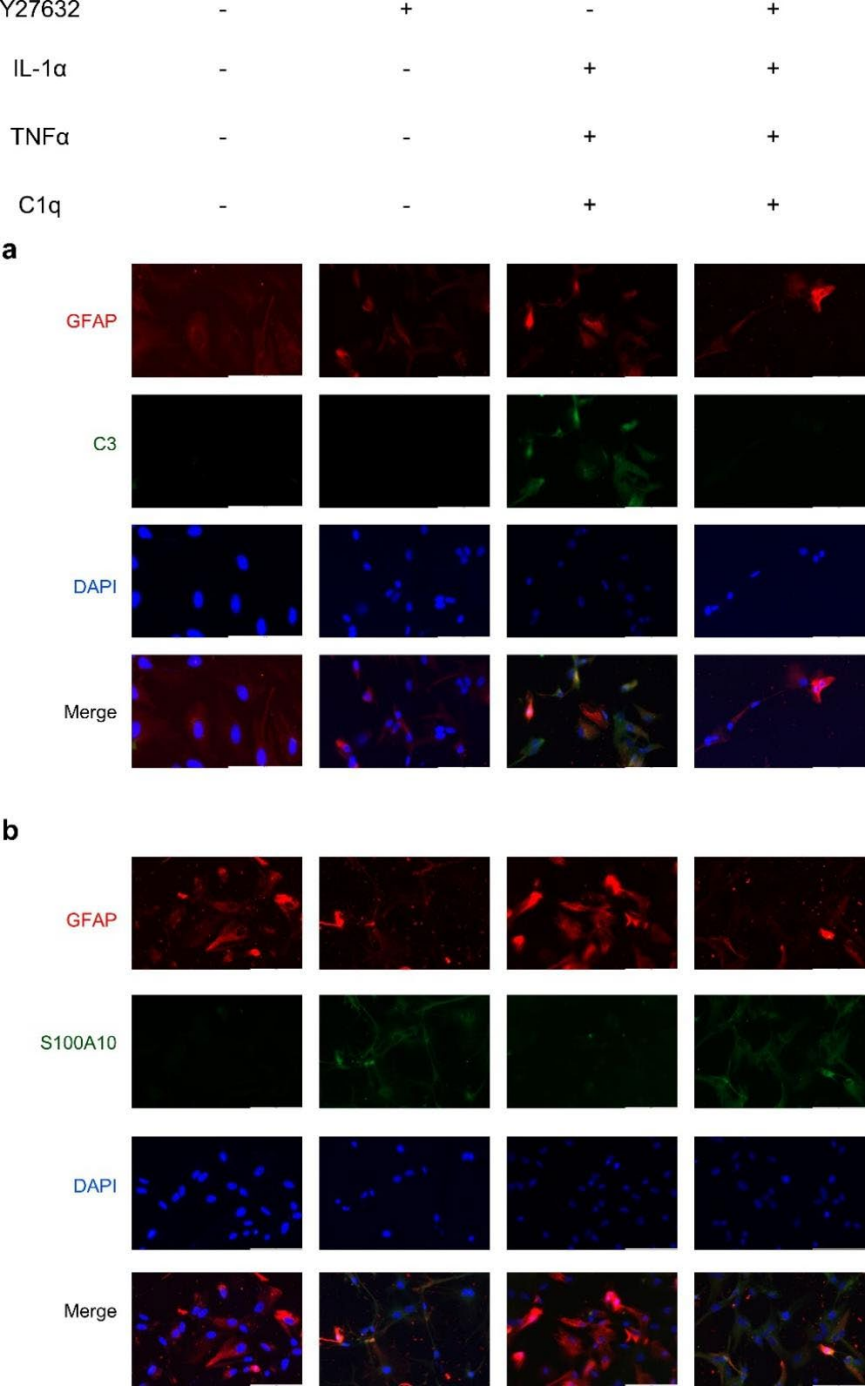
Y27632 transformed the naive astrocytes and the A1 reactive astrocytes into A2 reactive astrocytes. (**a**) Double fluorescent staining (GFAP in red, C3 in green) showed that astrocytes treated with IL-1α + TNFα + C1q were C3-positive, while the rest astrocytes were C3-negative. (**b**) Double fluorescent staining (GFAP in red, S100A10 in green) showed that astrocytes treated with Y27632 were S100A10-positvie, while the rest astrocytes were S100A10-nagative. DAPI stained all cell nuclei blue; Scale bars, 100μm

To systematically validate whether Y27632 stimulation effectively strengthens the astrocyte phenotype switch, we further investigated the changes of expression using a subset of characteristic markers for astrocyte subtypes that were treated for 72h under different treatment conditions. As shown in Fig.4, almost all naive astrocytes were GFAP^+^ /C3^−^/S100A10^−^, while the astrocytes treated with IL-1α + TNFα + C1q were GFAP^+^/C3^+^ (Fig.4a), a hallmark of A1 reactive astrocytes. With the addition of Y27632 into astrocyte cell cultures, cell immunostaining was GFAP^+^/S100A10^+^ (Fig.4b), suggesting that these astrocytes transformed into type A2 astrocytes. Notably, the astrocytes treated with IL-1α + TNFα + C1q following Y27632 treatment were GFAP^+^ /C3^−^/S100A10^+^ (Fig.4 right panel). These results demonstrated that Y27632 treatment the naive astrocytes and the A1 reactive astrocytes into A2 reactive astrocytes in vitro.

### Y27632 Promoted the Recovery of Neurological Function in SCI Rats

**Fig. 5:**
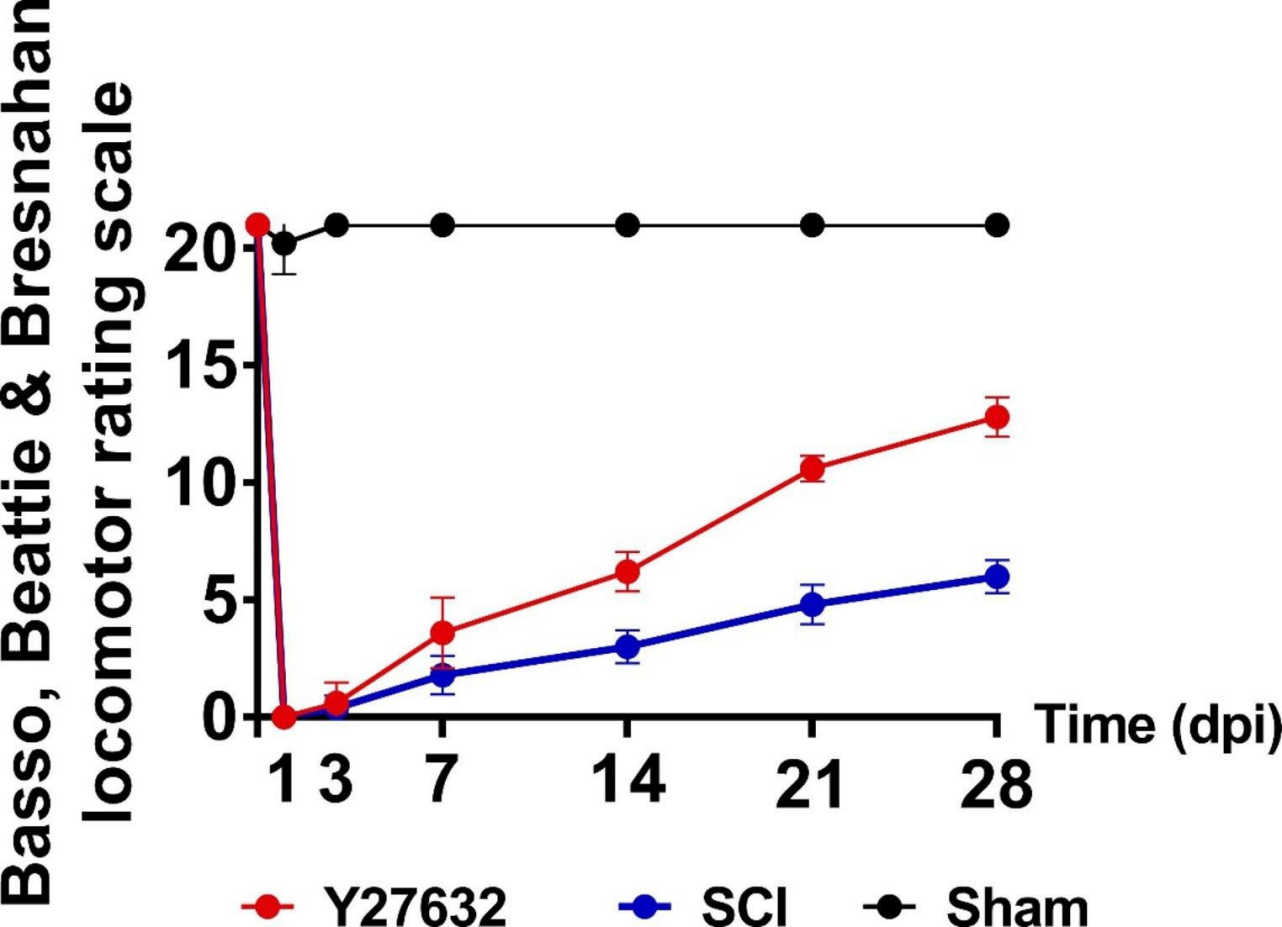
The BBB scores for rats in each group changed over time. There were no statistically significant differences in BBB scores among each recorded time point (1d, 3 d, 7 d, 14 d, 21 d and 28 d) in the sham group (*P* > 0.05) after operation. On day 28, the Y27632 group score was significantly higher than that of the SCI group (*P* < 0.01)

To evaluate the effects of Y27632 on SCI, an SCI rat model was established and treated with Y27632, followed by the evaluation of animal functional recovery. As shown in Fig.5, The BBB scores for the rats in each group changed over time. The sham group had a transient lower limb movement disorder on the day of operation and quickly recovered the next day. There were no statistically significant differences in the BBB scores among each recorded time point (1d, 3 d, 7 d, 14 d, 21 d and 28 d) in the sham group (*P* > 0.05) after the operation. On day 28, the scores of the sham, Y27632, and SCI groups were 21 ± 0, 12.80 ± 0.84, and 6.00 ± 0.71, respectively. In comparison, the scores of the SCI group and the Y27632 group were significantly lower than those of the sham group (SCI group vs. sham group, *P* < 0.001; Y27632 group vs. sham group, *P* < 0.001). More importantly, the Y27632 group score on day 28 was significantly higher than that of the SCI group (*P* < 0.01). These results suggest that Y27632 effectively enhances the recovery of neurological function in SCI rats.

### Y27632 Decreased the Number of A1 Astrocytes in the Spinal Cord of SCI Rats

**Fig. 6:**
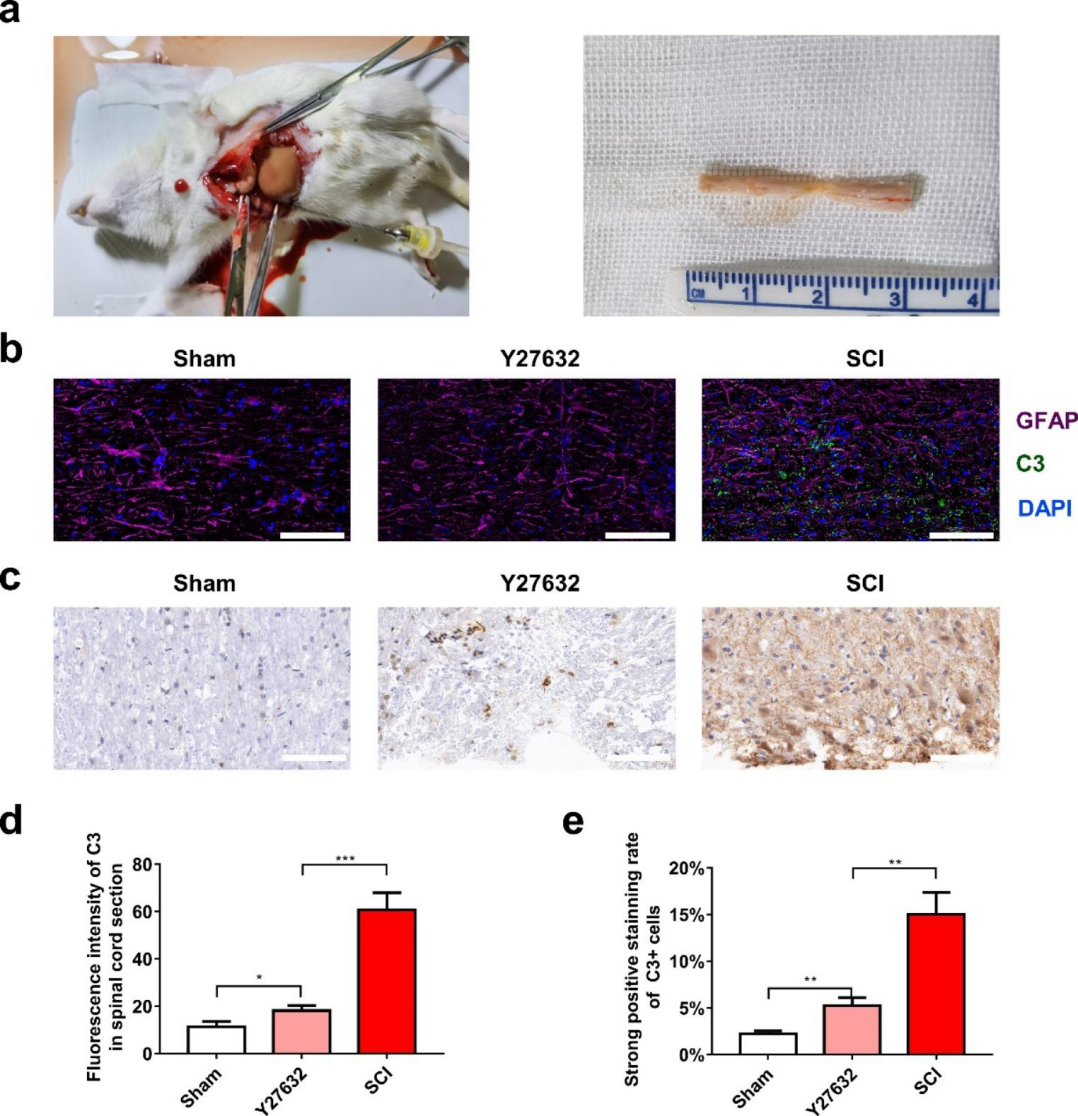
Y27632 promoted the recovery of neurological function by decreasing the number of A1 astrocytes in SCI rats. (**a**) Rats were anesthetized and perfused, then the spinal cord contained the damage area were harvested. (**b**) Double fluorescent staining(GFAP in purple, C3 in green)of spinal cord sections of the three group (Sham, Y27632 and SCI). (**c**) Immunochemical stained sections showed positive C3 cells in the three groups. (**d**) Quantification of the fluorescence intensity of C3. (**e**) The strong positive staining rate of C3 + cell in the three groups. Scale bars: 100μm

We next examined whether Y27632 contributes to the switch of astrocyte subtype that leads to the recovery of neurological function. The immunostaining of A1 astrocytes was thus conducted to examine the effect of Y27632 on A1 astrocytes after SCI. First, the rats were sacrificed one week after the SCI and the spinal cord tissues containing the damaged area were harvested as described in the method section (Fig.6a). Immunofluorescence double staining with anti-C3 and anti-GFAP antibodies showed that after the SCI, C3 was abundantly expressed within the injured area and was expressed at a relatively higher level in the cells compared to the sham group and the Y27632 treatment group (Fig.6b). Similar to C3 immunofluorescence, immunohistochemistry using anti-C3 antibodies also showed that after an SCI, almost all cells were significantly C3-positive (Fig.6c). Moreover, most cell populations exhibited a more pronounced C3 immunoreactivity than those in the sham and the Y27632 treatment groups (Fig.6c). Interestingly, quantitative analysis revealed that the administration of Y27632 significantly decreases the fluorescence intensity of C3 in spinal cord compared to the SCI group (*P* < 0.001, Fig.6d). Furthermore, the strong positive staining rate of C3^+^ cells in the Y27632 group was significantly lower than that in the SCI group (*P* < 0.01, Fig.6e).

### Y27632 Affected the Expression of C3 and NF-κB in Vitro

**Fig. 7:**
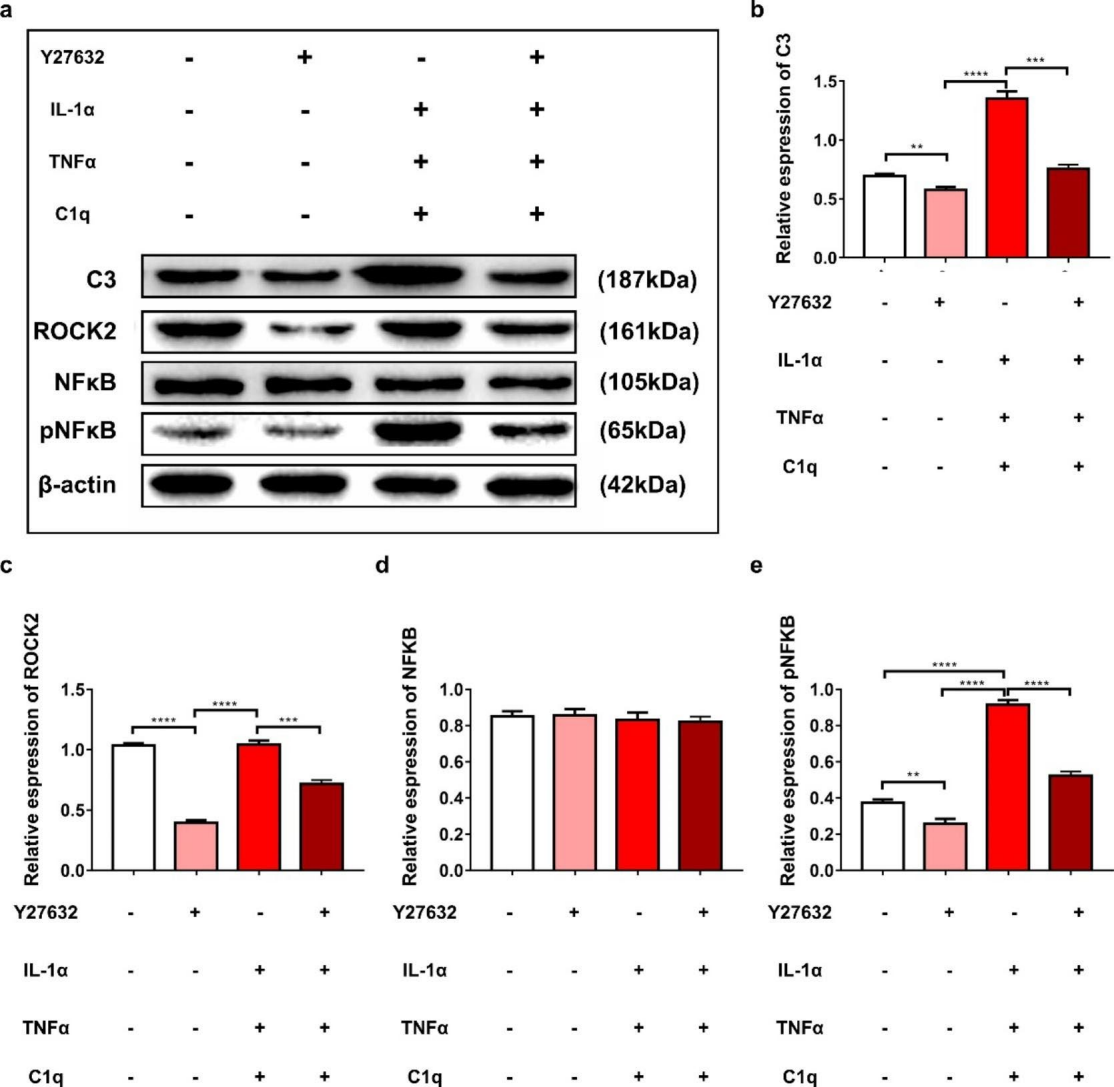
Possible molecular mechanism of Y27632 on the transformation of reactive astrocyte phenotypes in vitro. (**a**) Western blotting detected protein levels of C3, ROCK2, NF-κB, and phosphorylated NF-κB in astrocytes with different treatments. (**b-e**). Quantification of the relative expression of C3, ROCK2, NF-κB, and phosphorylated NF-κB of astrocytes in each group

The previous results of this study showed that Y27632 could transform the naive astrocytes and the A1 reactive astrocytes into A2 reactive astrocytes in vitro. Numerous studies have demonstrated that the downstream signal pathway of RhoA/ROCK, and NF-κB transcription activity impacts the complement C3 release of astrocytes. Therefore, we next ascertained the potential intracellular signaling mechanisms responsible for the promotive effects of Y27632 on the transformation process of astrocyte subtypes in vitro. As shown in Fig.7, the treatment of Y27632 markedly resulted in a decrease in ROCK2, phosphorylated NF-κB, and C3 levels within the astrocytes. In contrast, treatment with IL-1α + TNFα + C1q significantly elevated all the ROCK2 and phosphorylated NF-κB levels in the astrocytes, but no influence on ROCK2 (Fig.7a). Administration of Y27632 into the astrocytes treated with IL-1α + TNFα + C1q significantly reduced the elevation of all three molecule levels. Quantitative analysis revealed that C3 and the phosphorylated NF-κB levels in astrocytes treated by IL-1α + TNFα + C1q were significantly higher than that those in the control group and the Y27632 treatment group(IL-1α + TNFα + C1q vs. Y27632, *P* < 0.001; IL-1α + TNFα + C1q vs. Control, *P* < 0.001)(Fig.7b, c, d and e). Furthermore, Y27632 caused the downregulation of ROCK2 and the levels of phosphorylated NF-κB. These results imply that the phosphorylation levels of the NF-κB/C3 signaling cascade are actively involved in the transformation process of astrocytes that are stimulated via Y27632 treatment in vitro.

## Discussion

Astrocytes are ubiquitous and play an important role in the mammalian CNS. Astrocytes undergo a dramatic transformation called “reactive astrogliosis” and formed a glial scar after an acute SCI[36]. Reactive astrogliosis plays a dual roles in the recovery of neuronal function[37]. These opposite effects may depend on different phenotypes of reactive astrocytes, which are classified into either an A1 phenotype or an A2 phenotype[8]. These two reactive astrocyte phenotypes have strikingly different properties, one phenotype is beneficial for the recovery of SCI while the other is detrimental. Therefore, the transformation of astrocytes into a neuroprotective phenotype is likely critical for the recovery of neuronal function after an SCI.

Our present study showed that the recovery of neurological function after an SCI was significantly improved after being treated with the ROCK inhibitor Y27632. Our results are consistent with numerous other studies[20, 21, 38–41]. This study found that A1 phenotype reactive astrocytes were significantly reduced in SCI rats after Y27632 treatment. These results demonstrated that the neuroprotective effect of Y27632 may be attributed to its effects on the phenotype switch of astrocytes. Furthermore, we investigated the effects of Y27632 on astrocytes in vitro. The results showed that the naive astrocytes and the A1 phenotype astrocytes (GFAP^+^/C3^+^) after Y27632 treatment transform into A2 phenotype astrocytes (GFAP^+^/S100A10^+^). However, the molecular mechanisms underlying the effect of Y27632 on the switch of reactive astrocyte phenotypes remain unknown.

An increasing number of studies revealed that C3 is a specific marker of the reactive astrocyte A1 phenotype[8]. Furthermore, increases in C3 transcripts were positively associated with motor disability[23]. Because of this data, we also determined C3 expression after an SCI. Our study showed that the BBB scores improved significantly with the down-regulation of C3 expression and is consist with previous reports. Additionally, the expression of C3 decreased with the treatment of ROCK inhibitor Y27632 in vitro and in vivo. These results suggest that the C3 could be a downstream molecule of RhoA/ROCK signal pathway driving the effects of Y27632 on reactive astrocyte phenotypes.

NF-κB is a key transcription factor regulating inflammatory cytokine gene expression. Previous studies demonstrated that as the downstream intracellular signaling molecule of RhoA/ROCK, the NF-κB transcription activity was augmented by the activation of RhoA/ROCK[24–30]. Further research revealed that the activation of NF-κB increases the release of astrocytic complement C3 [22]. Therefore, we speculate that the inhibition of RhoA/ROCK elicits the switch of astrocyte subtypes from A1 to A2 through the collaboration of NF-κB/C3-mediated downstream signals to promote the recovery of SCI in rats.

In the present study, the pNF-κB levels were elevated by IL-1α + TNFα + C1q; therefore, increasing the expression of C3, a marker of A1 phenotype reactive astrocytes. The results suggest that the NF-κB/C3 signal pathway may be involved in the transformation of the A1 phenotype reactive astrocytes. Moreover, the expression of pNF-κB and C3 in the astrocytes was simultaneously downregulated after Y27632 treatment, inferring Y27632’s role in the transformation of reactive astrocytes from an A1 to an A2 phenotype. These results further revealed that the ROCK/NF-κB/C3 signal pathway is likely involved in mediating Y27632 promoting the recovery of SCI in rats.

Moreover, the RhoA/ROCK pathway played a negative regulatory role in the migration abilities of astrocytes. The cell migration process includes the protrusion of the leading edge, the formation of new adhesive structures at the front, the contraction of the cell, and the release of adhesions at the rear. Therefore, the alteration of cell morphology and cytoskeletal organization are associated with the actin polymerization during cell protrusion and traction caused by the contraction of actin filaments[42]. Previous studies demonstrated that after RhoA activation, ROCK regulates the contraction of the cytoskeleton by affecting the activity of myosin ATPase and both microtubules and microfilaments are involved during these processes[42–48]. In our present study, Y27632 induced a morphological shift of astrocytes from a flattened shape to a process-bearing shape, and significantly enhanced the migration ability of the transformed astrocytes that possess the process-bearing shape compared with the astrocytes that are flat shaped.

RhoA/ROCK controls a wide range of cellular processes, and it is challenging to reveal its comprehensive and specific molecular mechanism in astrocytes completely. Limitations of this study included (1) the lack of data on inhibitor or knockdown of NF-κB to confirm the activity of the NF-κB/C3 pathway regarding the promotion of Y27632 in the recovery of neurological function within a SCI rat model due to NF-κB playing a key role on numerous cellular activities; (2) the simultaneous effects of Y27632 on the morphology and phenotypes of astrocytes as well as playing a role in neuroprotection, however, the present study could not effectively distinguish these two effects independently; (3) the precise molecular mechanisms of the phenotypical shift induced by ROCK inhibitor Y27632 still needs further investigation.

Despite these shortcomings, to our knowledge, this is the first report demonstrating the possible molecular mechanism of the promoting effect of Y27632 on the recovery of neurological function in SCI via an astrocyte phenotypical shift. Importantly, Y27632 potentiates the functional recovery after SCI through an A1 to A2 phenotype astrocytes shift may open new avenues towards a potential therapy to repair an injured CNS.

## Conclusion

In summary, our study investigated the promotive effects of the ROCK inhibitor Y27632 on the recovery of neurological function in SCI, as well as the morphological and phenotypic shift of astrocytes. The present study demonstrates that the ROCK/NF-κB/C3 signal pathway may be involved in these cellular processes and account for the neuroprotective mechanism of action. Nevertheless, the relationship between the morphology and the phenotype of reactive astrocytes as well as the precise molecular mechanisms within SCI needs to be further explored in the future.

## Data Availability

The datasets generated during and/or analyzed during the current study are available from the corresponding author on reasonable request.
