# Correction: Rho Kinase Inhibitor Y27632 Improves Recovery After Spinal Cord Injury by Shifting Astrocyte Phenotype and Morphology via the ROCK/NF-?B/C3 Pathway

**DOI:** 10.1007/s11064-022-03764-0

**Published:** 2022-10-16

**Authors:** Yongyuan Zhang, Xiaohui Wang, Chao Jiang, Zhe Chen, Shuangyang Ni, Hong Fan, Zhiyuan Wang, Fang Tian, Jing An, Hao Yang, Dingjun Hao

**Affiliations:** 1 Xi’an Jiaotong University Health Science Center, 710000 Xi’an, China; 2 Department of Spine Surgery, Hong Hui Hospital, Xi’an Jiaotong University, 710054 Xi’an, China; 3 Xi’an Medical University, No.74 Han’guang North Road, Beilin District, Xi’an, Shaanxi Province China; 4 Department of Neurology, The Second Afliated Hospital of Xi’an Jiaotong University, 710004 Xi’an, China; 5 Translational Medicine Center, Hong Hui Hospital, Xi’an Jiaotong University, 710054 Xi’an, China

**Correction to: Neurochemical Research** 10.1007/s11064-022-03756-0

In the original online version of this article unfortunately contains errors, the authors would like to issue the following corrections:

1. Professor Hao Yang (yanghao.71_99@yahoo.com) should be incorporated as co-corresponding author.

2. The resolution of the Fig. 3 is replaced with high resolution. The high resolution of the Fig. 3 with the caption is given below.

Those changes do not affect the results of the study. We apologize to readers for those errors. The original version of this article is updated.

**Fig. 3:**
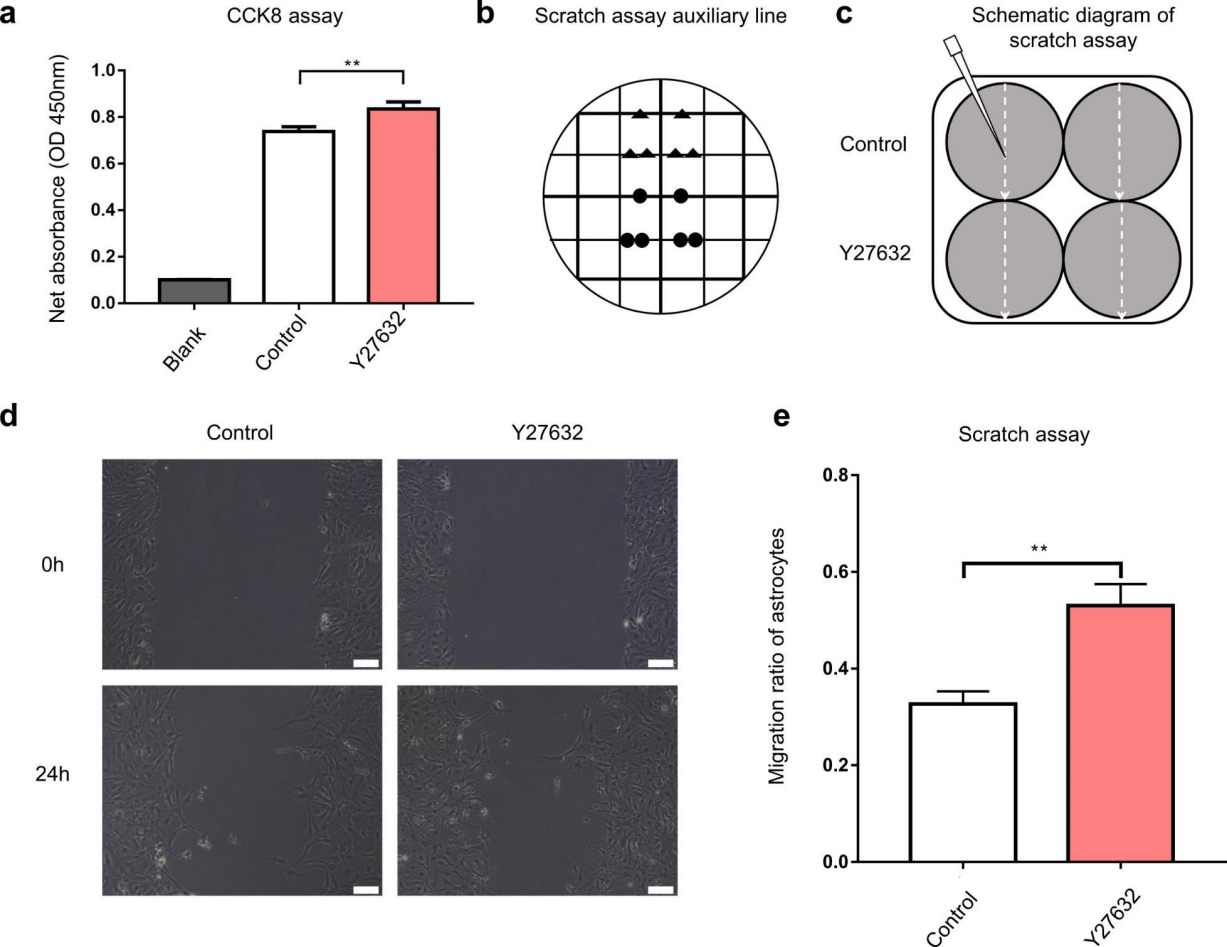
Y27632 increased the proliferation and migration abilities of the astrocytes. (**a**) The CCK8 assay showed that the Y27632 improved the proliferation of astrocytes. (**b**) The auxiliary lines assisted to the wound scratch. (**c**) A schematic diagram depicting the scratch assay. (**d**) The astrocytes were scraped by pipette tip (200 µL), and the final scratch areas (24 h later) were calculated. (**e**) The migration ratio of astrocytes treated with Y27632 was higher compared to the control group (*P* < 0.01). Scale bars, 100 μm

